# Synthesis of Moracin C and Its Derivatives with a 2-arylbenzofuran Motif and Evaluation of Their PCSK9 Inhibitory Effects in HepG2 Cells

**DOI:** 10.3390/molecules26051327

**Published:** 2021-03-02

**Authors:** Jagadeesh Nagarajappa Masagalli, Melanayakanakatte Kuberappa BasavanaGowda, Hee-Sung Chae, Won Jun Choi

**Affiliations:** College of Pharmacy and Integrated Research Institute for Drug Development, Dongguk University, Seoul 04620, Korea; mnjagadeesh123@gmail.com (J.N.M.); basavanagowdru@gmail.com (M.K.B.); chaeheesung83@gmail.com (H.-S.C.)

**Keywords:** proprotein convertase subtilisin/kexin type 9, low-density lipoprotein cholesterol, cardiovascular diseases, moracin compounds, structure activity relationships, HepG2 cell lines

## Abstract

Proprotein convertase subtilisin/kexin type 9 (PCSK9) is a key factor in several cardiovascular diseases, as it is responsible for the elevation of circulating low-density lipoprotein cholesterol (LDL-C) levels in blood plasma by direct interaction with the LDL receptor. The development of orally available drugs to inhibit this PCSK9-LDLR interaction is a highly desirable objective. Here, we report the synthesis of naturally occurring moracin compounds and their derivatives with a 2-arylbenzofuran motif to inhibit *PCSK9* expression. In addition, we discuss a short approach involving the three-step synthesis of moracin C and a divergent method to obtain various analogs from one starting material. Among the tested derivatives, compound **7** (97.1%) was identified as a more potent inhibitor of *PCSK9* expression in HepG2 cells than berberine (60.9%). These results provide a better understanding of the structure–activity relationships of moracin derivatives for the inhibition of PCSK9 expression in human hepatocytes.

## 1. Introduction

Cardiovascular diseases (CVDs) are one of the leading causes of death worldwide [1] and are caused by various cardiovascular factors [2], including the elevation of circulating low-density lipoprotein cholesterol (LDL-C) levels. Proprotein convertase subtilisin/kexin type 9 (PCSK9) plays an important role in regulating the circulating levels of LDL-C due to its ability to inhibit LDL receptor recycling in the liver [3]. The loss-of-function of PCSK9 in humans significantly lowers LDL-C levels and CVD-related morbidity, which indicates that PCSK9 could be a promising therapeutic target for the treatment of CVDs by lowering LDL-C levels [4]. Thus, PCSK9 inhibition has become the focus for the treatment of hypercholesterolemia.

Owing to the promise of their therapeutic potential, multiple PCSK9 monoclonal antibodies (mAbs) have been evaluated in clinical trials. Among these antibodies, two PCSK9 mAbs (alirocumab and evolocumab) have been approved by the United States Food and Drug Administration for the treatment of coronary heart disease. In addition, a small interfering RNA medication, inclisiran, which inhibits the synthesis of PCSK9, lowered LDL-C levels in patients with CVD and is in the process of being approved [5]. However, these drugs are costly and administered by injection only, which may restrict their clinical use. Therefore, an orally available small molecule targeting PCSK9 would be a highly desirable alternative therapeutic agent, based on its ease of administration and lower cost.

**Figure 1:**
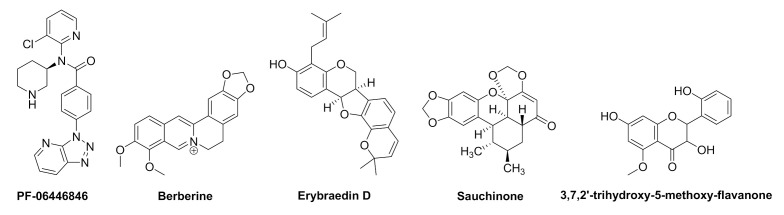
Small molecule inhibitors of proprotein convertase subtilisin/kexin type 9 (*PCSK9*) expression.

**Figure 2:**
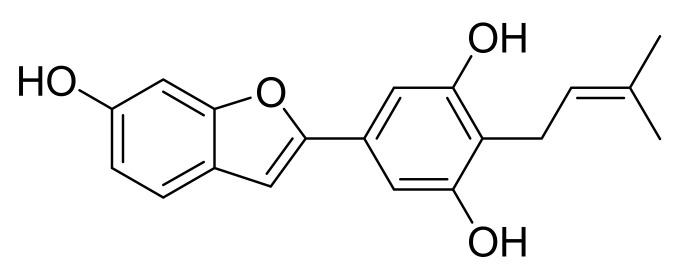
Structure of moracin C.

Small molecules targeting PCSK9—PF-06446846 analogs—identified by Pfizer have been found to lower the secretion of PCSK9 [6]. Natural products, such as berberine, erybraedin D, sauchinone, and 3,7,2′-trihydroxy-5-methoxy-flavanone, also exhibited potent inhibitory effects on PCSK9 levels in HepG2 cells [7,8,9] (Figure 1). Recently, moracin C (Figure 2), the bioactive ingredient of dried, immature *Morus alba* fruits, was reported to downregulate *PCSK9* expression in HepG2 cells [10].

Herein, we describe the preparation of a new series of 2-arylbenzofuran derivatives with PCSK9 inhibitory activity by chemical modifications of moracin C and the screening of a library of our synthesized small molecules using *PCSK9* expression in HepG2 cells as the readout.

## 2. Results and Discussion

### 2.1. Design and Synthesis of Moracin Derivatives

**Figure 3:**
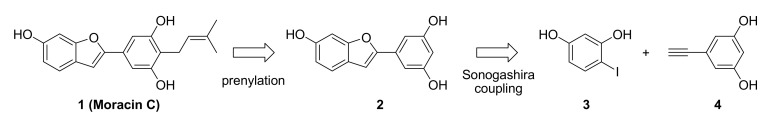
Retrosynthetic analysis of moracin C.

The retrosynthetic approach for moracin C is outlined in Figure 3. We envisaged that the aliphatic chain in moracin C (**1**) could be easily introduced by prenylation using *n*-BuLi and prenyl halide, and the construction of the benzofuran skeleton (**2**, moracin M) [11,12] could be achieved by the Sonogashira coupling of halobenzene-diol (**3**) with ethynylbenzene-diol (**4**).

**Scheme 1:**
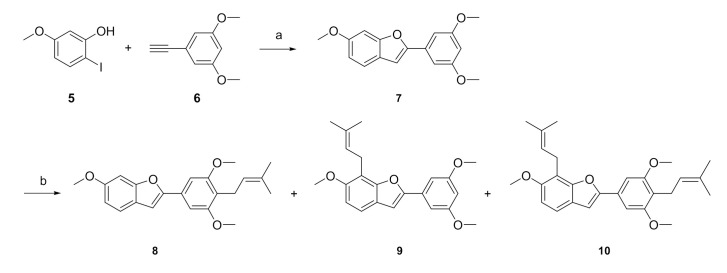
Reagents and Conditions: (**a**) PdCl_2_ (PPh_3_)_2_, CuI, TEA, DMF, 100 °C, 15 h and (**b**) 2.5-M *n*-BuLi in hexanes, 3,3-dimethylallyl bromide, cyclohexane, 0 °C to reflux, 2 h.

The formation of the 2-arylbenzofuran nucleus was easily accessible through the Sonogashira coupling reaction of 2-iodo-5-methoxyphenol **5** with 1-ethynyl-3,5-dimethoxybenzene **6** in the presence of PdCl_2_ (PPh_3_)_2_ and CuI in dimethylformamide (DMF). The yield of the Sonogashira product was higher (62%) than that reported previously [13]. Prenylation of the 2-arylbenzofuran nucleus **7** was carried out using *n*-BuLi to afford a mixture of three components: a 4′-prenylated compound **8** (25%), an isomeric 7-prenylated derivative **9** (12%), and a di-prenylated compound **10** (15%) (Scheme 1).

**Scheme 2:**
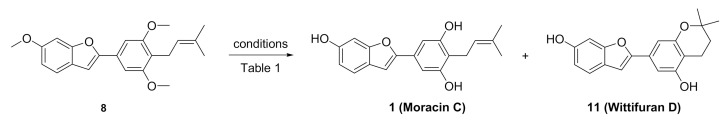
Demethylation of phenolic ether.

Several attempts were made for the demethylation of the methyl ether groups in compound **8** to give moracin C (**1**) by employing various conditions (such as BBr_3_, BCl_3_, AlCl_3_, and pyridinium *p*-toluenesulfonate (PPTS)) (Scheme 2) [14]. However, most attempts failed, resulting in the cyclized/chromane product **11** as the major product (Table 1).

The use of acidic reagents leads to a ring-fused/chromane formation due to the stability of the tertiary carbocation species. Interestingly, the cyclized/chromane compound **11** was found to be Wittifuran D, a natural compound present in the stem bark of *Morus wittiorum* (*Moraceae*) [15]. As a member of the library of moracin derivatives, Wittifuran D (**11**) was easily prepared by demethylation using a conventional reagent, BBr_3_, and this method also yielded moracin M (**2**) from **7**.

**Scheme 3:**
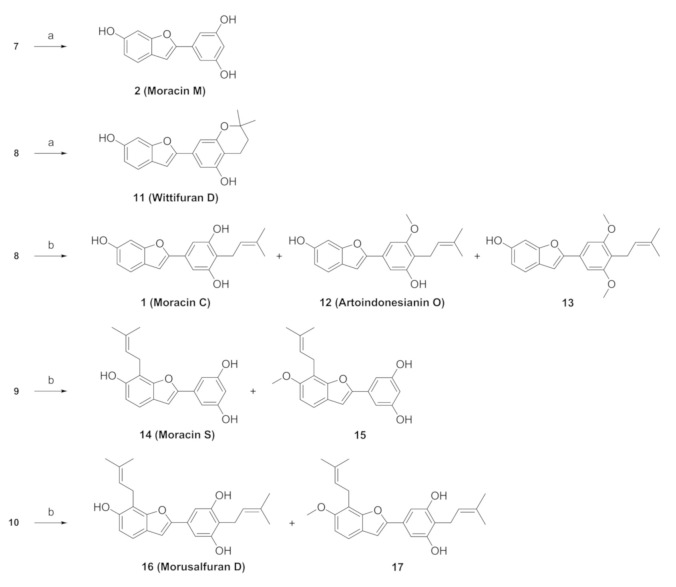
Reagents and conditions: (**a**) 1.0-M BBr_3_, CH_2_Cl_2_, −78 °C to room temperature (rt), 3 h and (**b**) 1-dodecanethiol, NMP, NaOH, 130 °C, 24 h.

Among the conditions presented in Table 1, a basic demethylation condition [16] using 1-dodecanethiol and NaOH in NMP met our objective in spite of the low yield and a product mixture consisting of **1** (30%), **12** (14%), and **13** (10%). Compound **12** was found to be a natural compound, Artoindonesianin O [17] (Scheme 3). The methyl group in the partially demethylated compound, Artoindonesianin O (**12**), is present at the ortho position to the prenyl group, which was confirmed by a two-dimensional nuclear magnetic resonance (NMR) analysis.

A similar method was applied to the intermediate compounds **9** and **10**, resulting in fully deprotected final products **14** (moracin S) [18] and **16** (morusalfuran D) [19] and two new partially deprotected moracin derivatives, **15** and **17** (Scheme 3).

### 2.2. Inhibitory Activity against PCSK9 mRNA Expression

**Figure 4:**
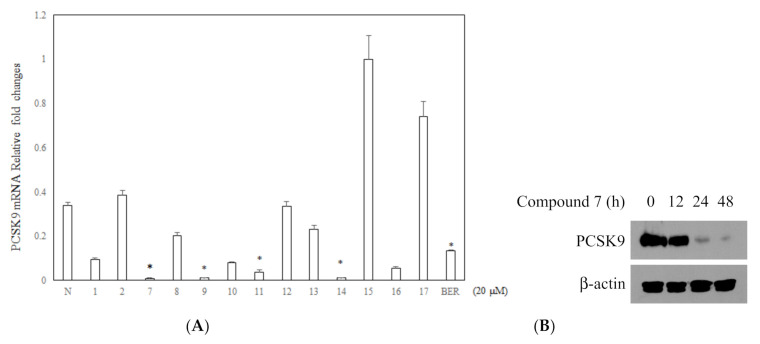
Identification of compound **7** as a strong inhibitor of PCSK9. (**A**) Evaluation of the PCSK9 mRNA level by quantitative RT-PCR. The expression of PCSK9 mRNA was measured in HepG2 cells after the treatment of compounds (20 μM) and berberine·HCl (BER; 20 μM) for 24 h. Mean values of three independent experiments are shown, and the asterisk (*) indicates *p* < 0.05. (**B**) Western blot analysis indicates that compound **7** inhibited the expression of PCSK9 in HepG2 cells.

The inhibitory effects of a series of synthesized Moracin C and its derivatives on PCSK9 mRNA was measured by quantitative RT-PCR using human hepatoma HepG2 cells (Figure 4A).

Among them, **1**, **7**, **9**, **11**, and **14** were found to significantly inhibit the expression of PCSK9 mRNA, whose percent inhibitory activities were 44.9%, 97.1%, 96.7%, 88.5%, and 96.3% (berberine 60.9%), respectively. However, other compounds failed to exhibit suppressive effects. In particular, compound **7**, the protected Moracin C precursor without a prenyl group, exhibited the strongest inhibitory effect. To confirm whether compound **7** can downregulate the expression of PCSK9, we exposed compound **7** to HepG2 cells, conducted a Western blot analysis, and found that compound **7** suppressed the expression of PCSK9 protein (Figure 4B).

In conclusion, the present approach to synthesize moracin C in three steps was found to be more efficient than those reported previously in terms of both yield and time [20,21]. The exclusive scale-up synthesis of the other natural products presented herein will be reported in the near future. Various prenylated 2-arylbenzofuran derivatives could exert more persistent and enhanced biological effects than moracin C.

The results of the present study provide a better understanding of the structure–activity relationships of moracin derivatives for the inhibition of *PCSK9* expression in human hepatocytes. This information would be useful for the rational design of new drugs for hypercholesterolemia.

## 3. Materials and Methods

### 3.1. Figures, Tables, and Schemes

Except where noted, all the materials were purchased from commercial suppliers (Sigma Aldrich, Darmstadt, Germany; Tokyo Chemical Industy Co., Ltd. Tokyo, Japan; Alfa, Ward Hill, MA, USA; Combi-blocks, San Diego, CA, USA) and were used without further purification. All reactions were routinely carried out under an inert atmosphere of dried nitrogen. ^1^H-NMR spectra (CDCl_3_, CD_3_OD, and (CD_3_)_2_CO) were recorded on a Varian (400 MHz) spectrometer (Varian Medical Systems, Inc., Palo Alto, CA, USA). The ^1^H-NMR data are reported as peak multiplicities: s for singlet, d for doublet, dd for doublet of doublets, t for triplet, q for quartet, bs for broad singlet, and m for multiplet. ^13^C-NMR spectra (CDCl_3_, CD_3_OD, and (CD_3_)_2_CO) were recorded on a Varian (100 MHz) spectrometer (Palo Alto, CA, USA). The chemical shifts are reported as parts per million (δ) relative to the solvent peak, with the coupling constants in hertz (Hz). Infrared spectra were recorded on Fourier-transform infrared spectroscopy (FTIR, NICOLET-iS5, Waltham, MA, USA). Reactions were monitored with Thin-layer chromatography (TLC, Merck precoated 60 F254 plates, Darmstadt, Germany). Spots were detected by viewing under a UV light and colorizing with charring after dipping in KMnO_4_ solution. Column chromatography was performed on silica gel 60 (230–400 mesh Kieselgel 60). Purity of the final compound was determined using reversed-phase high-pressure liquid chromatography (RP-HPLC) performed on a Waters Corp. HPLC system equipped with an ultraviolet (UV) detector at 254 nm operating at 25 °C. The HPLC employed a YMC hydrosphere C18 (HS-302) column (5-μm particle size and 12-nm pore size) with a diameter of 4.6 mm and length of 150 mm at a flow rate of 1.0 mL/min. Mobile phase: (A) H_2_O containing 0.05% trifluoroacetic acid and (B) CH_3_CN. Method I: a gradient of 25% B to 100% B in 30 min and Method II: a gradient of 50% B to 100% B in 30 min. (The purity data are provided in Supplementary Materials.) The purity of all tested compounds was > 95% purity using Method I and Method II. The mass spectra were recorded using LRMS (electron ionization Mass spectrometry (MS)) obtained on a Shimadzu-2020 or using HRMS (electrospray ionization MS) obtained on a G2 Quadrupole time-of-flight (QTOF) mass spectrometer.

### 3.2. Cell Culture, Drugs and Chemicals

HepG2 human hepatocellular cells were obtained from the Korea Research Institute of Bioscience and Biotechnology (KRIBB, Daejeon, Korea) and grown in Eagle’s Minimum Essential Media (EMEM) containing 10% fetal bovine serum and 100-U/mL penicillin/streptomycin (Pen/Strep). Cells were cultured in a humidified incubator with 5% CO_2_ at 37 °C. EMEM and Pen/Strep were purchased from Hyclone (Logan, UT, USA). Bovine serum albumin (BSA) was purchased from Sigma-Aldrich (St. Louis, MO, USA). Antibodies against PCSK9 and *β*-actin were purchased from Abcam (Cambridge, MA, USA).

### 3.3. Quantitative Real-Time RT-PCR

Total RNA was isolated using a Trizol RNA extraction kit (Thermo Fisher Korea, Seoul, Korea) according to the manufacturer’s instructions. Briefly, total RNA (1 μg) was converted to Complementary DNA (cDNA) by reverse transcriptase (200 unit) and oligo- deoxythymidine triphosphate (dT) primer (500 ng) in 50-mM Tris-HCl (pH 8.3), 75-mM KCl, 3-mM MgCl_2_, 10-mM dithiothreitol (DTT), and 1-mM deoxyribose Nucleoside Triphosphates (dNTPs) at 42 °C for 1 h. The reaction was terminated by incubating the solution at 70 °C for 15 min; after which, 1-µl cDNA mixture was mixed for PCR amplification. PCR reaction was performed using 1-μL cDNA and 9-μL master mix containing iQ SYBR Green Supermix (Bio-Rad, Hercules, CA, USA), forward primer (5 pmol), and reverse primer (5 pmol) in a CFX384 Real-Time PCR Detection System (Bio-Rad, Hercules, CA, USA). PCR reaction was as follow: 3 min at 95 °C, followed by 40 cycles of 95 °C for 10 sec, 55 °C for 30 sec, and 72 °C for 2 min. The fluorescence signal generated by SYBR Green I DNA dye was measured. PCR primers were obtained from Bioneer (Daejeon, Korea), and the specificity of the primers was confirmed using the melting curve analysis. Data were collected and managed by CFX Manager Software (Bio-Rad, Hercules, CA, USA). The sequences of the PCR primer sets were as follows: human PCSK9 (Forward: GGTACTGACCCCCAACCTG and Reverse: CCGAGTGTGCTGACCATACA) and human Glyceraldehyde 3-phosphate dehydrogenase (GAPDH) (Forward: GAAGGTGAAGGTCGGAGTCA and Reverse: AATGAAGGGGTCATTGATGG).

### 3.4. Statistical Analysis

The data from the experiments were expressed as the mean ± S.E.M. The level of statistical significance was determined by analysis of variance (ANOVA), followed by Dunnett′s *t*-test for multiple comparisons. *P*-values less than 0.05 were regarded as statistically significant.

### 3.5. Western Blot Analysis

Sample were ground by pestle and incubated with 200-μL RIPA buffer (50-mM Tris-HCl at pH 8.0, 150-mM NaCl, 1% NP-40, 0.5% sodium deoxycholate, and protease inhibitors cocktail) for 1 h on ice. Lysates were collected by centrifugation, and protein concentration was measured by the BCA Protein Assay Kit (Thermo Fisher, Pittsburgh, PA, USA). Equal amounts of lysates were resolved by SDS-PAGE and transferred to the Polyvinylidene fluoride (PVDF) membrane. The membrane was incubated in blocking buffer (5% skim milk in 1x PBS-0.1% Tween-20; PBST) for 1 h and hybridized with the appropriate primary antibody in 1x PBS containing 3% bovine serum albumin (BSA) overnight at 4 °C. After washing three times with 1x PBST for 30 min, the membrane was hybridized with the appropriate Horseradish peroxidase (HRP)-conjugated secondary antibody (Cell Signaling Technology, Danvers, MA, USA) for 1 h at room temperature and washed three times with 1x PBST solution for 30 min. The membrane was visualized using an enhanced chemiluminescence (ECL) detection system.

### 3.6. Synthetic Methods

*2-(3,5-Dimethoxyphenyl)-6-methoxybenzofuran* (**7**): To a stirred solution of 2-iodo-5-methoxyphenol **5** (1 g, 4.2 mmol) in dry DMF (8 mL) under N_2_ atmosphere were added successively PdCl_2_(PPh_3_)_2_ (148 mg, 0.21 mmol), CuI (27 mg, 0.25 mmol), and Et_3_N (1.8 mL, 12.6 mmol). After 5 min, 1-ethynyl-3,5-dimethoxybenzene **6** (1 g, 6.3 mmol) was added, and the mixture was stirred at 80 °C for 15 h. The reaction mixture was cooled to room temperature and acidified with dilute HCl and extracted with ethyl acetate. The organic layer was washed with water, then dried over MgSO_4_, filtered, and evaporated. The residue was purified by silica gel column chromatography (Hex:EtOAc = 9:1, *v*/*v*) to afford compound **7** (750 mg, 62% yield) as a pale yellow solid: ^1^H-NMR (400 MHz, CDCl_3_) δ 7.40 (d, *J* = 8.8 Hz, 1H), 7.05 (d, *J* = 2.4 Hz, 1H), 6.95 (d, *J* = 2.4 Hz, 2H), 6.91 (d, *J* = 0.8 Hz, 1H), 6.85 (dd, *J* = 2.0 and 8.4 Hz, 1H), 6.43 (t, *J* = 2.0 Hz, 1H), 3.84 (s, 3H), 3.83 (s, 6H); ^13^C-NMR (100 MHz, CDCl_3_) δ 161.07, 158.15, 155.82, 154.90, 132.43, 122.42, 121.06, 112.03, 102.50, 101.71, 100.54, 95.80, 55.69, 55.67, 55.44, 55.42; IR (neat) 2935, 2829, 1598, 1444, 1204, 1137, 810 cm*^−^*^1^; LCMS Electrospray ionization (ESI) *m*/*z* calcd for C_17_H_17_O_4_ [M + H]^+^: 285.1127, found: 285.1139; Purity 96.21% (determined by RP-HPLC, method I, *t*_R_ = 21.64 min).

#### 3.6.1. Procedure for Prenylation 

*n*-Butyllithium (1.6 M solution in hexane, 1.64 mL, 2.63 mmol) was added dropwise to a stirred solution of **7** (500 mg, 1.75 mmol) in absolute cyclohexane (15 mL) at 0 °C under N_2_ atmosphere. The reaction mixture was heated at 60 °C for 30 min. After cooling to room temperature, 3,3-dimethylallylbromide (0.3 mL, 2.63 mmol) was added dropwise, and the resulting mixture was stirred at 60 °C for 2 h [22]. The reaction mixture was cooled and poured into a saturated solution of sodium bicarbonate, extracted with ethyl acetate, washed with water, dried over MgSO_4_, filtered, and evaporated. The crude residue was purified by column chromatography (Hex:EtOAc = 9.5:0.5, *v*/*v*) to get the products **8**, **9**, and **10**.

*6-Methoxy-2-(3,5-dimethoxy-4-(3-methylbut-2-enyl)phenyl)benzofuran* (**8**): 154 mg (25% yield) as a white solid: ^1^H-NMR (400 MHz, CDCl_3_) δ 7.42 (d, *J* = 8.4 Hz, 1H), 7.08 (bs, 1H), 6.98 (s, 2H), 6.91 (s, 1H), 6.86 (dd, *J* = 1.6 and 8.8 Hz, 1H), 5.20 (t, *J* = 7.2 Hz, 1H), 3.90 (s, 6H), 3.87 (s, 3H), 3.36 (d, *J* = 6.8 Hz, 2H), 1.78 (s, 3H), 1.67 (s, 3H); ^13^C-NMR (100 MHz, CDCl_3_) δ 158.22, 157.92, 155.69, 155.53, 131.42, 129.24, 122.58, 122.50, 120.83, 118.73, 111.85, 100.74, 100.30, 95.84, 55.87, 55.73, 25.87, 22.32, 17.75; IR (neat) 2840, 1567, 1489, 1405, 1170, 1106, 818 cm^−1^; LCMS (ESI) *m*/*z* calcd for C_22_H_25_O_4_ [M + H]^+^: 353.1753, found: 353.1768; Purity 98.30% (determined by RP-HPLC, method II, *t*_R_ = 23.73 min).

*6-Methoxy-2-(3,5-dimethoxyphenyl)-7-(3-methylbut-2-enyl)benzofuran* (**9**): 74 mg (12% yield) as a pale yellow solid: ^1^H-NMR (400 MHz, CDCl_3_) δ 7.33 (d, *J* = 8.8 Hz, 1H), 6.99 (d, *J* = 2.4 Hz, 2H), 6.93 (s, 1H), 6.86 (d, *J* = 8.8 Hz, 1H), 6.44 (t, *J* = 2.4 Hz, 1H), 5.40 (t, *J* = 1.6 Hz, 1H), 3.91 (s, 3H), 3.87 (s, 6H), 3.65 (d, *J* = 7.6 Hz, 2H), 1.91 (s, 3H), 1.69 (s, 3H); ^13^C-NMR (100 MHz, CDCl_3_) δ 161.03, 155.11, 154.94, 154.24, 132.65, 131.72, 122.80, 122.09, 117.89, 113.70, 108.05, 102.64, 101.88, 101.86, 100.42, 56.72, 56.69, 55.44, 55.42, 25.82, 22.93, 17.84; IR (neat) 2999, 2934, 2831, 1601, 148, 1283, 1200, 1147, 1026, 815 cm^−1^; LCMS (ESI) *m*/*z* calcd for C_22_H_25_O_4_ [M + H]^+^: 353.1753, found: 353.1770; Purity 97.66% (determined by RP-HPLC, method I, *t*_R_ = 26.89 min).

*6-Methoxy-2-(3,5-dimethoxy-4-(3-methylbut-2-enyl)phenyl)-7-(3-methylbut-2-enyl)benzofuran* (**10**): 110 mg (15% yield) as a colourless gummy liquid: ^1^H-NMR (400 MHz, CDCl_3_) δ 7.30 (d, *J* = 8.8 Hz, 1H), 7.01 (s, 2H), 6.90 (s, 1H), 6.85 (d, *J* = 8.4 Hz, 1H), 5.41 (quint, *J* = 1.6 and 8.0 Hz, 1H), 5.20 (quint, *J* = 1.6 and 6.8 Hz, 1H), 3.90 (s, 9H), 3.66 (d, *J* = 7.6 Hz, 2H), 3.37 (d, *J* = 7.2 Hz, 2H), 1.93 (s, 3H), 1.78 (s, 3H), 1.69 (s, 3H), 1.67 (s, 3H); ^13^C-NMR (100 MHz, CDCl_3_) δ 158.19, 155.58, 154.92, 154.12, 131.58, 131.38, 129.48, 122.99, 122.56, 122.14, 118.72, 117.67, 113.58, 107.90, 100.88, 100.40, 56.70, 55.79, 25.86, 25.84, 22.98, 22.33, 17.80, 17.76; IR (neat) 2997, 2899, 2832, 1570, 1494, 1408, 1237, 1162, 1112, 1078, 802, 743 cm^−1^; LCMS (ESI) *m*/*z* calcd for C_27_H_33_O_4_ [M + H]^+^: 421.2379, found: 421.2372; Purity 98.41% (determined by RP-HPLC, method II, *t*_R_ = 30.66 min).

*7-(6-Hydroxybenzofuran-2-yl)-2,2-dimethylchroman-5-ol*, Wittifuran D (**11**): To a stirred solution of **8** (100 mg, 0.28 mmol) in dry DCM (4 mL) at −78 °C under nitrogen conditions was added dropwise 1-M BBr_3_ solution in CH_2_Cl_2_ (1.4 mL, 1.42 mmol) and stirred at the same temperature for 2 h, then room temperature for 1 h. After completion of the reaction, the excess BBr_3_ was quenched by adding ice water at 0 °C. The reaction mixture was warmed to room temperature and extracted with ethyl acetate 2 times. The combined organic layer was dried over MgSO_4_, filtered, and evaporated. The residue was purified by silica gel column chromatography (Hex:EtOAc = 7:3, *v*/*v*) to afford compound **11** (50 mg, 60% yield) as a white solid: ^1^H-NMR (400 MHz, CDCl_3_) δ 7.36 (d, *J* = 8.4 Hz, 1H), 6.97 (br, 1H), 6.89 (s, 1H), 6.81 (s, 2H), 6.75 (d, *J* = 8.8 Hz, 1H), 4.96 (s, 2H), 2.69 (t, *J* = 6.8 Hz, 2H), 1.84 (t, *J* = 6.4 Hz, 2H), 1.36 (s, 6H); ^13^C-NMR (100 MHz, CDCl_3_) δ 155.54, 155.24, 155.04, 154.13, 153.42, 129.72, 122.90, 121.08, 111.94, 108.85, 106.17, 102.42, 100.91, 98.23, 74.32, 32.04, 26.65, 17.00; IR (neat) 3391, 2969, 1634, 1561, 1492, 1422, 1207, 1120, 972 cm^−1^; LCMS (ESI) *m*/*z* calcd for C_19_H_19_O_4_ [M + H]^+^: 311.1283, found: 311.1284; Purity 96.20% (determined by RP-HPLC, method I, *t*_R_ = 15.93 min).

*5-(6-Hydroxybenzofuran-2-yl)benzene-1,3-diol*, Moracin M (**2**): 45 mg (70% yield from **7**) as a white solid: ^1^H-NMR (400 MHz, CD_3_OD) δ 7.33 (d, *J* = 8.8 Hz, 1H), 6.89 (s, 1H), 6.88 (d, *J* = 2.0 Hz, 1H), 6.74 (d, *J* = 2.4 Hz, 2H), 6.71 (dd, *J* = 2.0 and 8.4 Hz, 1H), 6.23 (t, *J* = 2.4 Hz, 1H); ^13^C-NMR (100 MHz, CD_3_OD) δ 158.52, 155.80, 155.41, 154.67, 132.37, 121.60, 120.58, 111.80, 102.46, 102.05, 100.78, 97.01; IR (neat) 3522, 3248, 1609, 1424, 1357, 1279, 1131, 997, 955, 802 cm^−1^; LCMS (ESI) *m*/*z* calcd for C_14_H_11_O_4_ [M + H]^+^: 243.0651, found: 243.0649; Purity 96.42% (determined by RP-HPLC, method I, *t*_R_ = 7.46 min).

#### 3.6.2. General Procedure for Demethylation

Powdered NaOH (254 mg, 6.3 mmol) was added to a resealable tube containing aryl methyl ethers (**8**, **9**, and **10**) (0.7 mmol). The tube was evacuated with vacuum and filled back with N_2_ gas. Anhydrous NMP (2 mL) was added to the mixture, followed by an addition of 1-dodecanethiol (1 mL, 5.0 mmol). The reaction mixture was stirred at 130 °C until the aryl methyl ether was consumed, as monitored by TLC. After reaction completion, the mixture was allowed to cool to room temperature, then acidified with 1-N HCl and diluted with (Ethyl Acetate) EtOAc. The aqueous phase was extracted with EtOAc, and the combined organic layers were washed with water and brine, dried over MgSO_4_, filtered, and evaporated. The residue was purified by column chromatography (Hex:EtOAc = 6:4, *v*/*v*) to afford compounds **1** and **12–17**.

*5-(6-Hydroxy benzofuran-2-yl)-2-(3-methylbut-2-enyl) benzene-1,3-diol*, Moracin C (**1**): 70 mg (30% yield) as a white solid: ^1^H-NMR (400 MHz, CD_3_OD) δ 7.33 (d, *J* = 8.8 Hz, 1H), 6.88 (br, 1H), 6.83 (s, 1H), 6.78 (s, 2H), 6.72 (dd, *J* = 2.0 and 8.4 Hz, 1H), 5.26 (t, *J* = 6.4 Hz, 1H), 3.32 (d, *J* = 6.4 Hz, 2H), 1.78 (s, 3H), 1.67 (s, 3H); ^13^C-NMR (100 MHz, CD_3_OD) δ 156.08, 155.68, 155.15, 155.05, 129.89, 128.79, 122.92, 121.75, 120.35, 115.40, 111.64, 102.33, 99.83, 96.97, 24.58, 21.89, 16.51; ^1^H-NMR (400 MHz, (CD_3_)_2_CO) δ 7.36 (d, *J* = 8.4 Hz, 1H), 6.94 (d, *J* = 2.0 Hz, 1H), 6.89 (s, 2H), 6.88 (d, *J* = 0.4 Hz, 1H), 6.78 (dd, *J* = 2.0 and 8.4 Hz, 1H), 5.32-5.28 (m, 1H), 3.37 (d, *J* = 6.8 Hz, 2H), 1.76 (s, 3H), 1.63 (s, 3H); ^13^C-NMR (100 MHz, (CD_3_)_2_CO) δ 156.24, 155.74, 155.54, 154.86, 130.08, 128.96, 123.06, 121.75, 120.90, 115.34, 112.14, 102.90, 100.52, 97.42, 24.99, 21.18, 17.00; IR (neat) 3332, 2916, 1612, 1489, 1436, 1039, 980, 830 cm^−1^; LCMS (ESI) *m*/*z* calcd for C_19_H_19_O_4_ [M + H]^+^: 311.1283, found: 311.1288; Purity 98.17% (determined by RP-HPLC, method I, *t*_R_ = 14.32 min).

*2-(3-Hydroxy-5-methoxy-4-(3-methylbut-2-enyl)phenyl)benzofuran-6-ol*, Artoindonesianin O (**12**): 33 mg (14% yield) as a white solid: ^1^H-NMR (400 MHz, CD_3_OD) δ 7.33 (d, *J* = 8.4 Hz, 1H), 6.92 (s, 1H), 6.92 (s, 3H), 6.73 (dd, *J* = 1.6 and 8.4 Hz, 1H), 5.20 (t, *J* = 6.8 Hz, 1H), 3.86 (s, 3H), 3.31 (d, *J* = 6.0 Hz, 2H), 1.76 (s, 3H), 1.65 (s, 3H); ^13^C-NMR (100 MHz, CD_3_OD) δ 160.14, 157.23, 157.16, 156.75, 156.43, 131.45, 130.55, 124.25, 123.23, 121.91, 118.13, 113.20, 105.33, 101.66, 99.65, 98.50, 56.18, 26.02, 23.25, 17.93; IR (neat) 3282, 2921, 1614, 1504, 1437, 1413, 1359, 1299, 1282, 1164, 1141, 1083, 970, 941, 838, 774 cm^−1^; LCMS (ESI) *m*/*z* calcd for C_20_H_21_O_4_ [M + H]^+^: 325.1440, found: 325.1445; Purity 99.58% (determined by RP-HPLC, method I, *t*_R_ = 18.95 min).

*2-(3,5-Dimethoxy-4-(3-methylbut-2-enyl)phenyl)benzofuran-6-ol* (**13**): 24 mg (10% yield) as a half white solid: ^1^H-NMR (400 MHz, CD_3_OD) δ 7.38 (d, *J* = 8.4 Hz, 1H), 7.02 (bs, 1H), 6.98 (s, 2H), 6.90 (s, 1H), 6.77 (dd, *J* = 2.0 and 8.2 Hz, 1H), 5.20 (t, *J* = 7.2 Hz, 1H), 4.91 (s, 1H), 5.91 (s, 6H), 3.37 (d, *J* = 6.8 Hz, 2H), 1.78 (s, 1H), 1.67 (s, 3H); ^13^C-NMR (100 MHz, CD_3_OD) δ 158.26, 155.74, 155.60, 153.49, 131.45, 129.18, 122.97, 122.52, 120.98, 118.89, 111.96, 100.74, 100.43, 98.27, 55.91, 25.86, 22.35, 17.76; IR (neat) 3095, 2921, 2848, 1747, 1569, 1409, 1221, 1104, 962, 827 cm^−1^; LCMS (ESI) *m*/*z* calcd for C_21_H_23_O_4_ [M + H]^+^: 339.1596, found: 339.1599; Purity 99.15% (determined by RP-HPLC, method I, *t*_R_ = 23.80 min).

*5-(6-Hyroxy-7-(3-methylbut-2-enyl)benzofuran-2-yl)benzene-1,3-diol*, Moracin S (**14**): 35 mg (15% yield) as a white solid: ^1^H-NMR (400 MHz, CD_3_OD) δ 7.15 (d, *J* = 8.4 Hz, 1H), 6.87 (s, 1H), 6.76 (d, *J* = 1.6 H, 2H), 6.70 (d, *J* = 8.0 Hz, 1H), 6.21 (t, *J* = 2.0 Hz, 1H), 5.40 (t, *J* = 7.6 Hz, 1H), 3.58 (d, *J* = 7.2 Hz, 2H), 1.88 (s, 3H), 1.68 (s, 3H); ^13^C-NMR (100 MHz, CD_3_OD) δ 158.50, 154.44, 154.36, 152.37, 132.59, 130.89, 122.05, 121.43, 117.46, 111.61, 111.17, 102.39, 101.88, 101.06, 24.54, 22.20, 16.68; IR (neat) 3338, 3192, 1645, 1480, 1422, 1134, 1036, 807 cm^−1^; LCMS (ESI) *m*/*z* calcd for C_19_H_19_O_4_ [M + H]^+^: 311.1283, found: 311.1284; Purity 98.86% (determined by RP-HPLC, method I, *t*_R_ = 12.77 min).

*5-(6-Methoxy-7-(3-methylbut-2-enyl)benzofuran-2-yl)benzene-1,3-diol* (**15**): 33 mg (14% yield) as a white solid: ^1^H-NMR (400 MHz, CD_3_OD) δ 7.35 (d, *J* = 8.8 Hz, 1H), 6.88 (bs, 1H), 6.74 (dd, *J* = 2.0 and 9.0 Hz, 1H), 6.70-6.69 (m, 2H), 6.46 (d, *J* = 2.0 Hz, 1H), 5.09 (t, *J* = 5.2 Hz, 1H), 3.43 (d, *J* = 6.4 Hz, 2H), 2.96 (s, 3H), 1.65 (s, 6H); ^13^C-NMR (100 MHz, CD_3_OD) δ 158.94, 155.90, 155.64, 155.26, 154.43, 131.44, 130.10, 124.04, 121.49, 120.52, 119.29, 111.67, 106.59, 104.41, 98.83, 96.95, 54.65, 25.13, 24.45, 16.60; IR (neat) 2921, 2850, 1605, 1424, 1141, 1115, 1042, 979, 817 cm^−1^; LCMS (ESI) *m*/*z* calcd for C_20_H_21_O_4_ [M + H]^+^: 325.1440, found: 325.1444; Purity 95.38% (determined by RP-HPLC, method I, *t*_R_ = 17.18 min).

*5-(6-Hyroxy-7-(3-methylbut-2-enyl)benzofuran-2-yl)-2-(3-methylbut-2-enyl)benzene-1,3-diol*, Morusalfuran D (**16**): 35 mg (15% yield) as a white solid: ^1^H-NMR (400 MHz, CD_3_OD) δ 7.13 (d, *J* = 8.0 Hz, 1H), 6.80 (s, 2H), 6.79 (s, 1H), 6.69 (d, *J* = 8.4 Hz, 1H), 5.40 (t, *J* = 7.2 Hz, 1H), 5.25 (t, *J* = 7.2 Hz, 1H), 3.58 (d, *J* = 7.6 Hz, 2H), 3.30 (d, *J* = 7.2 Hz, 2H), 1.86 (s, 3H), 1.76 (s, 3H), 1.67 (s, 3H), 1.65 (s, 3H); ^13^C-NMR (100 MHz, CD_3_OD) δ 156.06, 154.83, 154.25, 152.13, 130.95, 129.89, 129.06, 122.97, 122.12, 121.61, 117.26, 115.29, 111.54, 111.23, 102.37, 100.20, 24.61, 24.54, 22.22, 21.92, 16.73, 16.55; IR (neat) 3377, 2930, 1617, 1413, 1022, cm^−1^; LCMS (ESI) *m*/*z* calcd for C_24_H_27_O_4_ [M + H]^+^: 379.1909, found: 379.1916; Purity 97.69% (determined by RP-HPLC, method I, *t*_R_ = 18.93 min).

*5-(6-Methoxy-7-(3-methylbut-2-enyl)benzofuran-2-yl)-2-(3-methylbut-2-enyl)benzene-1,3-diol* (**17**): 33 mg (14% yield) as a white solid: ^1^H-NMR (400 MHz, CDCl_3_) δ 7.24 (d, *J* = 7.6 Hz, 1H), 6.94 (s, 1H), 6.92 (s, 1H), 6.87 (s, 1H), 6.76 (d, *J* = 8.4 Hz, 1H), 5.42 (t, *J* = 7.6 Hz, 1H), 5.37 (s, 1H), 5.29 (s, 1H), 5.25 (t, *J* = 7.2 Hz, 1H), 3.89 (s, 3H), 3.71 (d, *J* = 7.2 Hz, 2H), 3.43 (d, *J* = 7.2 Hz, 2H), 1.09 (s, 3H), 1.82 (s, 3H), 1.77 (s, 3H), 1.75 (s, 3H); ^13^C-NMR (100 MHz, CD_3_OD) δ 158.62, 155.69, 154.70, 154.32, 152.23, 130.77, 129.97, 129.31, 122.82, 122.14, 121.63, 117.36, 116.53, 111.61, 111.18, 103.86, 100.51, 98.13, 54.69, 24.55, 24.54, 22.25, 21.79, 16.68, 16.47; IR (neat) 3273, 2913, 1620, 1492, 1416, 1223, 1165, 1092, 807 cm^−1^; LCMS (ESI) *m*/*z* calcd for C_25_H_29_O_4_ [M + H]^+^: 393.2066, found: 393.2063; Purity 99.73% (determined by RP-HPLC, method I, *t*_R_ = 23.94 min).

## Figures and Tables

**Table 1:** Reaction conditions for demethylation *.

Entries	Conditions	Additives	Solvents	Temp (°C)	Time (h)	Yields (%)	
						1	11
1	BBr_3_	-	CH_2_Cl_2_	−78 to rt	3	-	60
2	BCl_3_	-	CH_2_Cl_2_	−78	4	-	42
3	AlCl_3_	Thiourea	CH_2_Cl_2_	50	4	-	-
4	*n*-BuLi	Diphenyl phosphine	THF	−78 to reflux	36	10	-
5	Sodium mercaptan	-	DMF	60	5	-	-
6	PPTS	-	DMF	120	15 min	-	35
7	Pyridine·HCl	-	DMF	200	3	-	40
**8**	**NaOH**	**1-Dodecane thiol**	**NMP**	**130**	**24**	**30**	**-**

* THF: tetrahydrofuran; NMP: *N*-methylpyrrolidone; Bold aims to highlight the good result.

## Data Availability

The data presented in this study are available on request from the corresponding author.

## Supplementary Materials

The supplementary materials are available online.

## Sample Availability

Samples of the compounds are available from the corresponding author.
