# Supplementary material for: Synthesis of Moracin C and Its Derivatives with a 2-arylbenzofuran Motif and Evaluation of Their PCSK9 Inhibitory Effects in HepG2 Cells
Source: Molecules. 2021 Mar 2;26(5):1327. doi: 10.3390/molecules26051327 (PMC7958322; doi:10.3390/molecules26051327)
Supplement: Supplementary file 1 [file molecules-26-01327-s001.pdf]

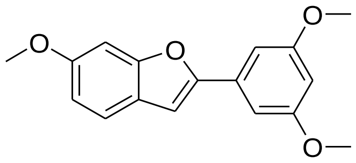

7

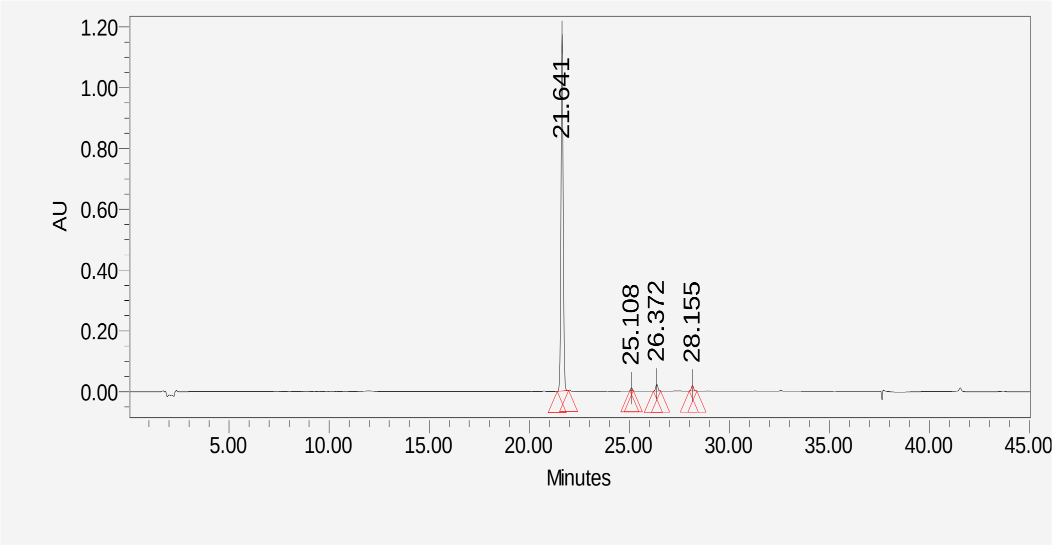

Name	Retention Time	Area	% Area	Height
1	21.641	8860460	96.21	1172085
2	25.108	47229	0.51	8525
3	26.372	161614	1.75	22081
4	28.155	140133	1.52	18776

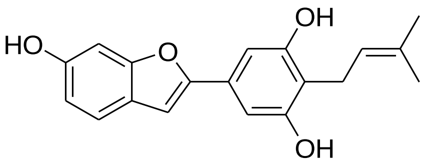

1 (Moracin C)

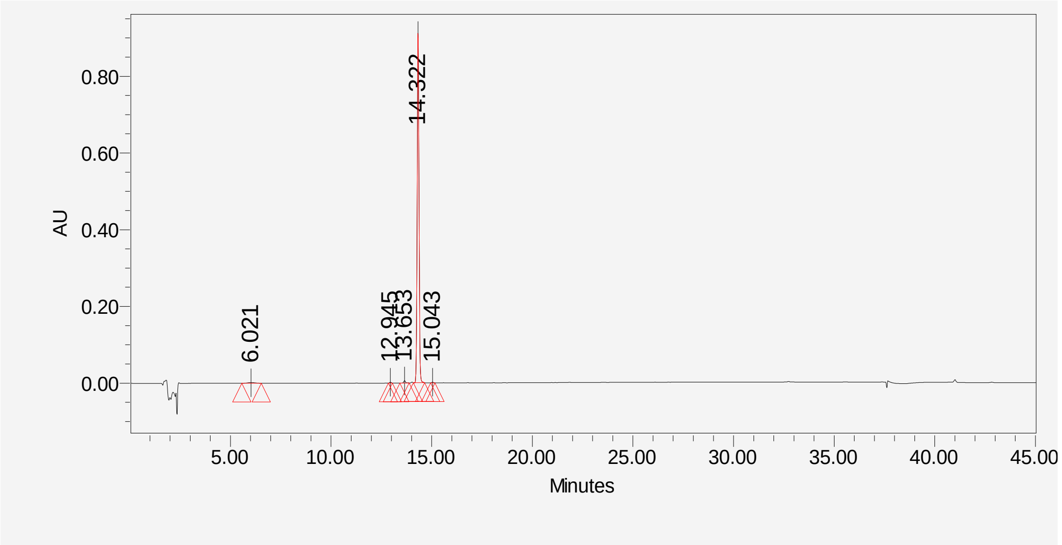

Name	Retention Time	Area	% Area	Height
1	6.021	45601	0.78	1675
2	12.945	13349	0.23	2369
3	13.653	36726	0.63	6010
4	14.322	5744480	98.17	915448
5	15.043	11506	0.2	1981

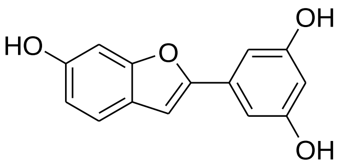

2 (Moracin M)

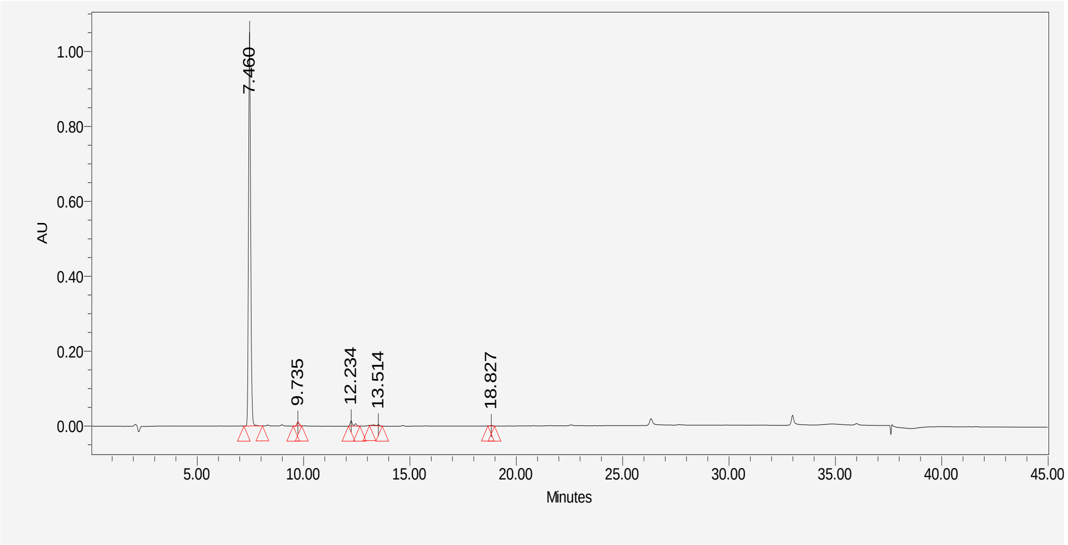

Name	Retention Time	Area	% Area	Height
1	7.46	7297333	96.42	1046473
2	9.735	79939	1.06	10850
3	12.234	136443	1.8	14263
4	13.514	45370	0.6	3221
5	18.827	9505	0.13	1515

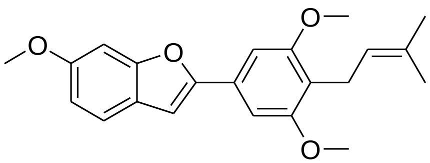

8

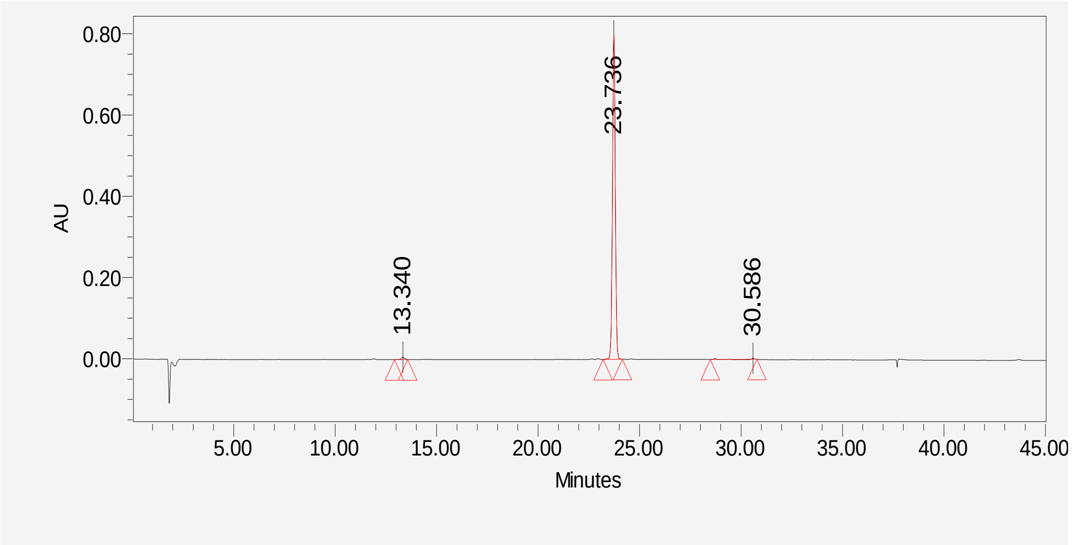

Name	Retention Time	Area	% Area	Height
1	13.34	56121	0.72	6035
2	23.736	7704571	98.3	798454
3	30.586	77094	0.98	2755

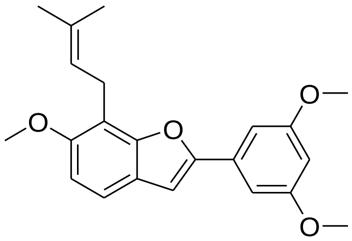

9

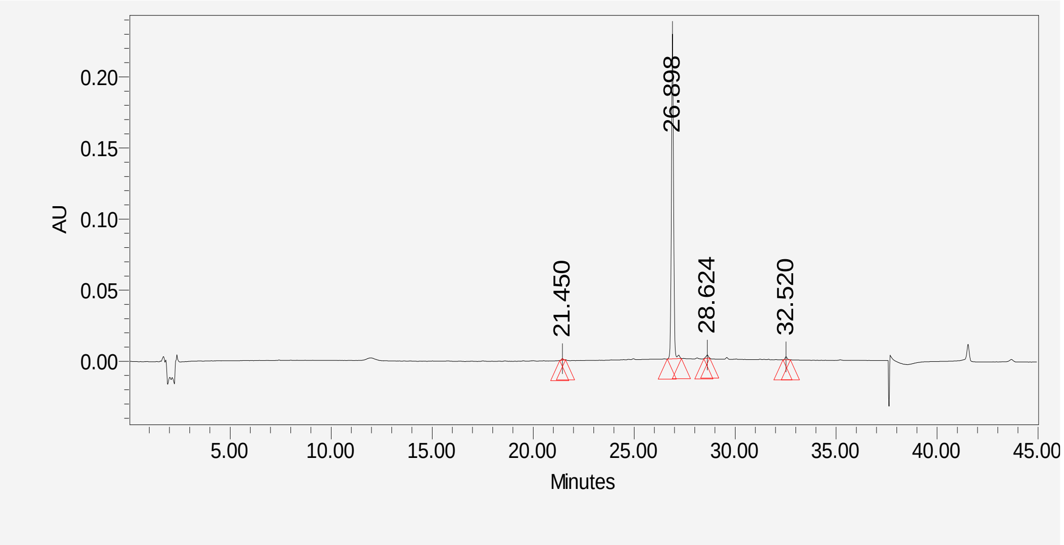

Name	Retention Time	Area	% Area	Height
1	21.45	6714	0.39	1089
2	26.898	1673659	97.66	227948
3	28.624	19409	1.13	2374
4	32.52	14044	0.82	2080

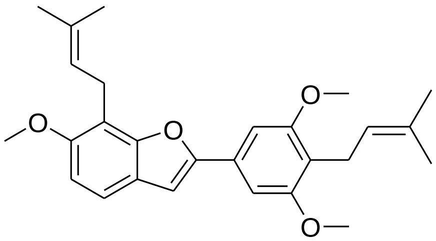

10

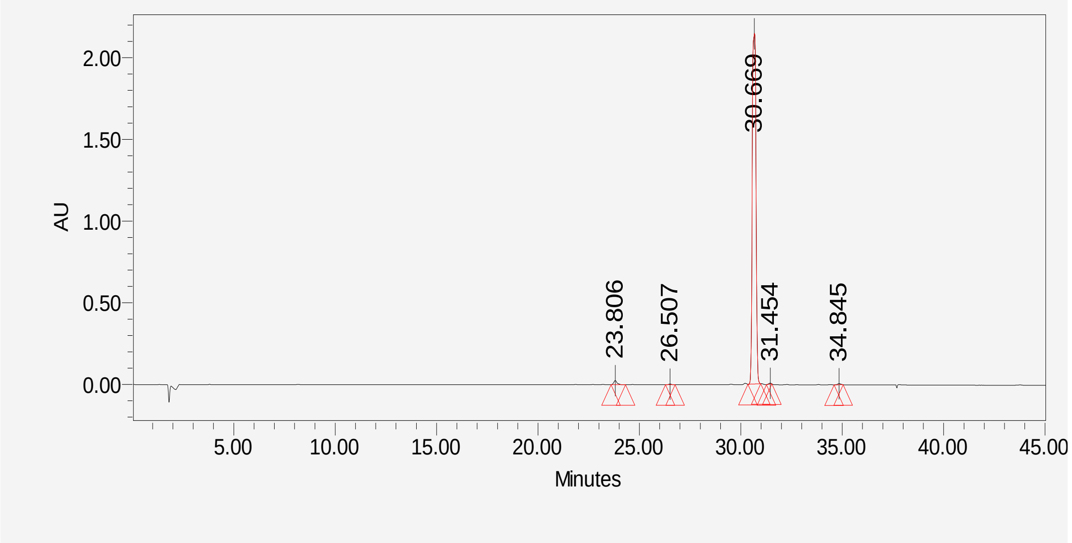

Name	Retention Time	Area	% Area	Height
1	23.806	280519	1.01	25698
2	26.507	37074	0.13	4154
3	30.669	27320898	98.41	2154094
4	31.454	45746	0.16	5119
5	34.845	78957	0.28	7441

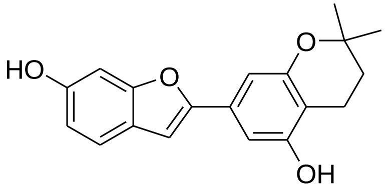

11 (Wittifuran D)

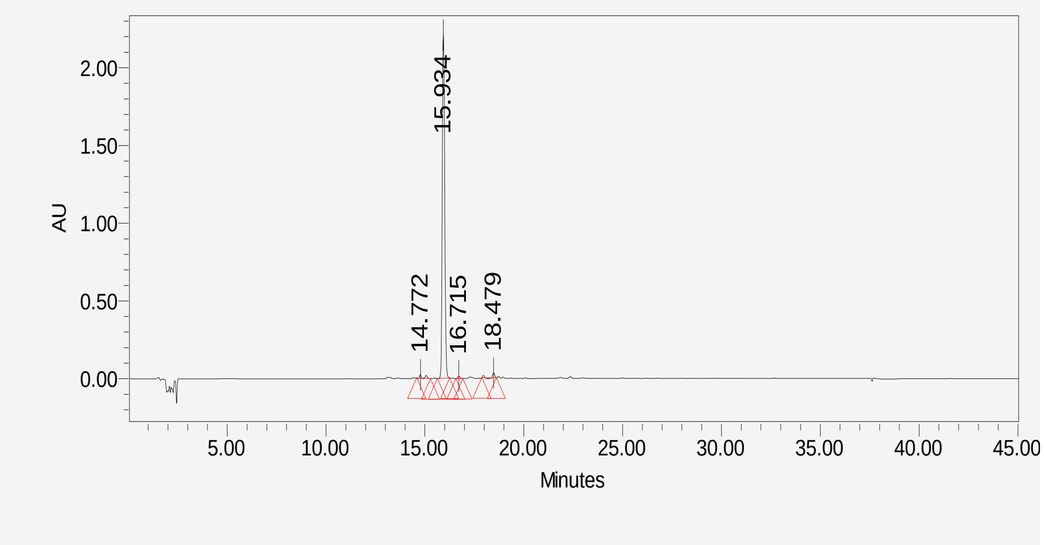

Name	Retention Time	Area	% Area	Height
1	14.772	268589	1.34	22601
2	15.934	1.9E+07	96.2	2219882
3	16.715	114771	0.57	16294
4	18.479	379790	1.89	31885

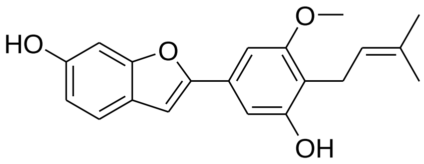

12 (Artoindonesianin O)

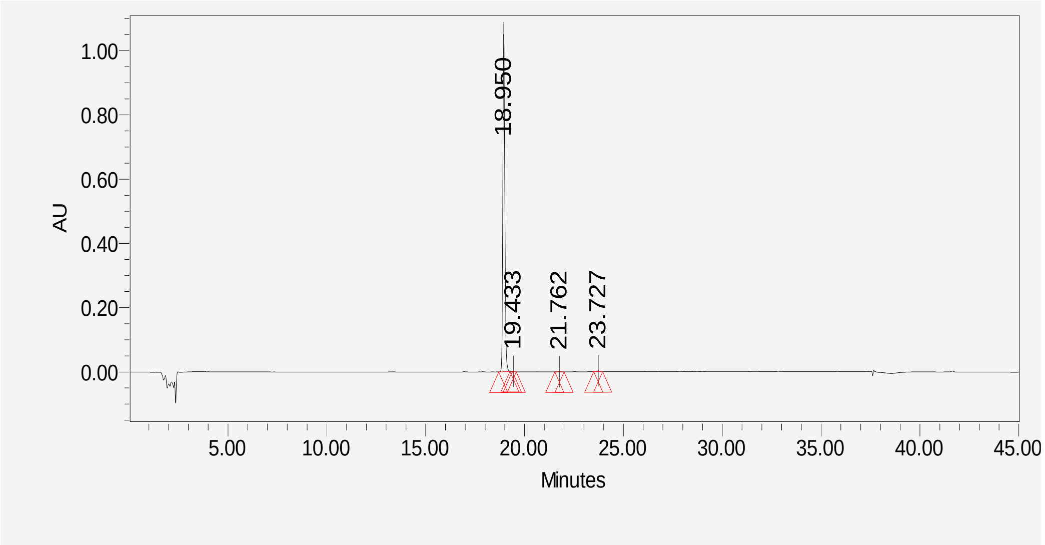

Name	Retention Time	Area	% Area	Height
1	18.95	7388637	99.58	1049565
2	19.433	5529	0.07	1089
3	21.762	5946	0.08	610
4	23.727	19435	0.26	2883

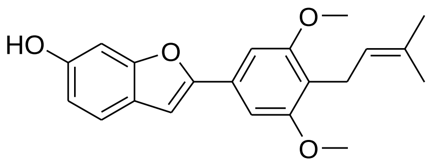

13

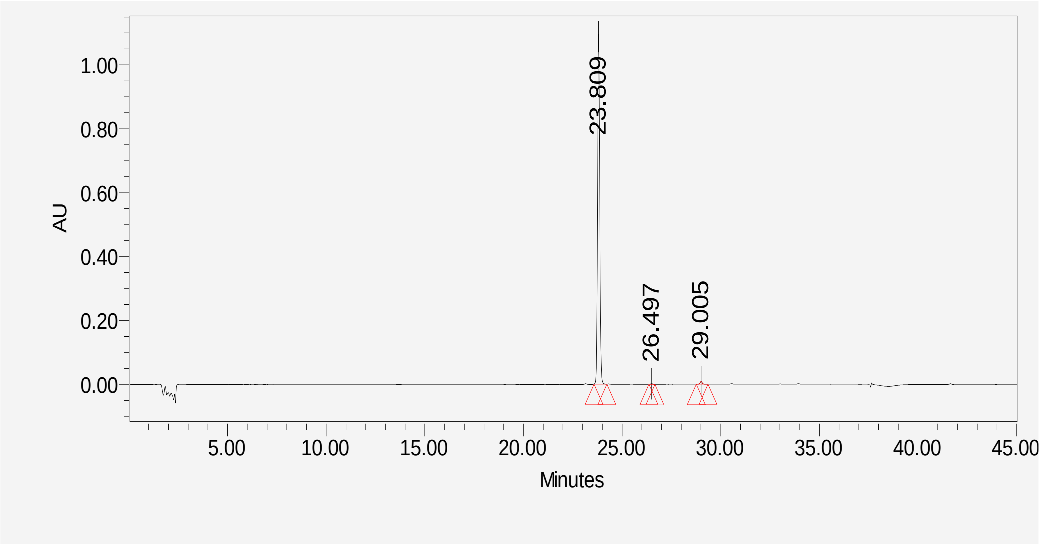

Name	Retention Time	Area	% Area	Height
1	23.809	8255924	99.15	1091136
2	26.497	12597	0.15	1899
3	29.005	58281	0.7	8218

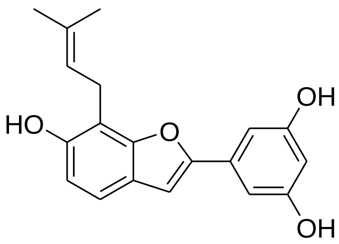

14 (Moracin S)

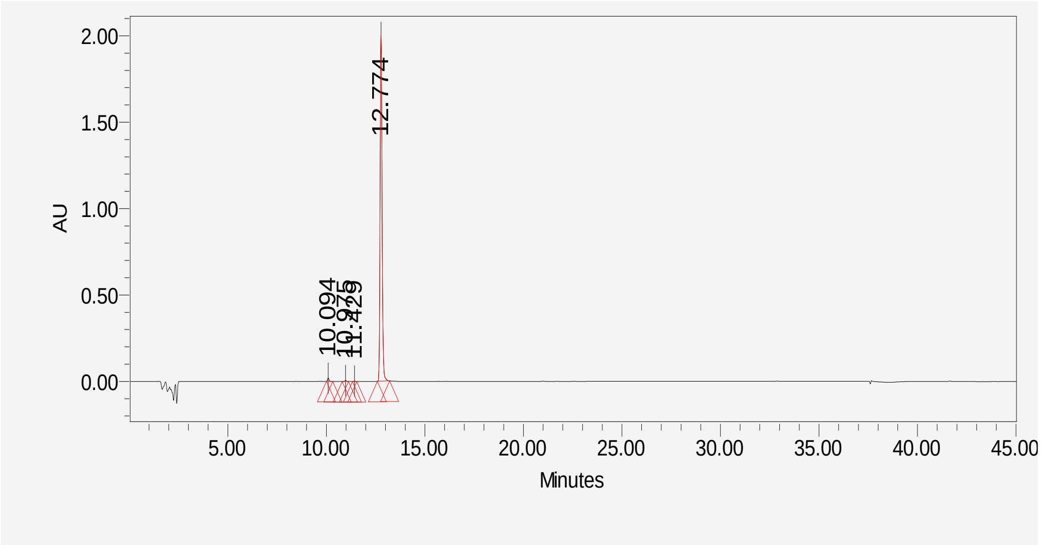

Name	Retention Time	Area	% Area	Height
1	10.094	109389	0.76	18113
2	10.975	34763	0.24	5655
3	11.429	18498	0.13	3005
4	12.774	1.4E+07	98.86	2004336

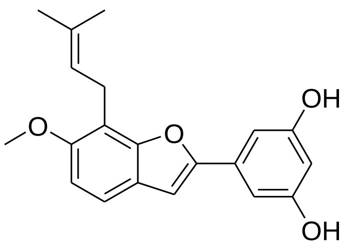

15

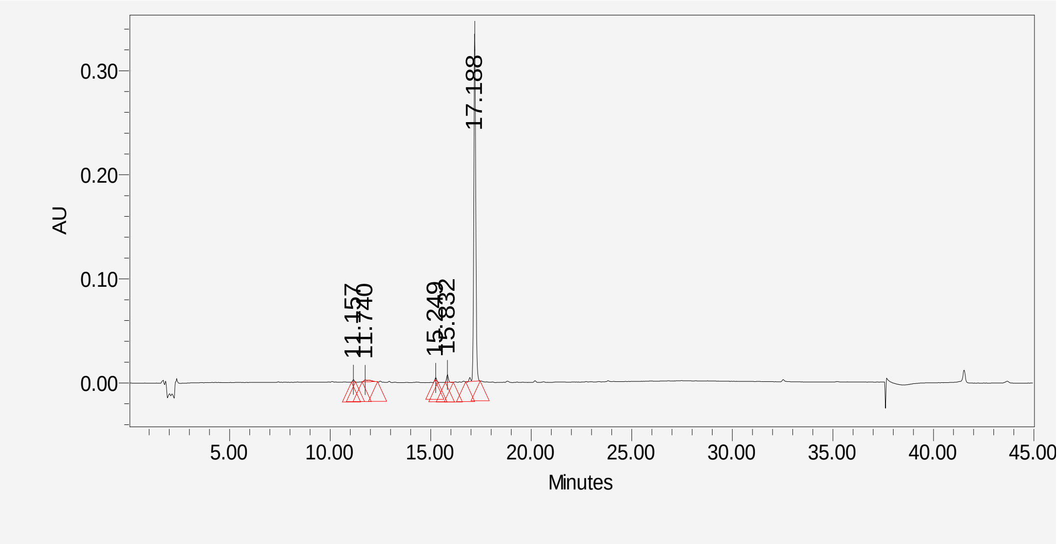

Name	Retention Time	Area	% Area	Height
1	11.157	12744	0.54	2275
2	11.74	38196	1.61	1852
3	15.249	12034	0.51	2558
4	15.832	46259	1.95	7146
5	17.188	2257592	95.38	332006

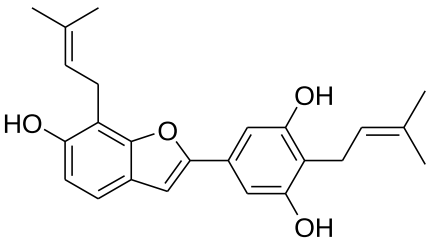

16 (Morusalfuran D)

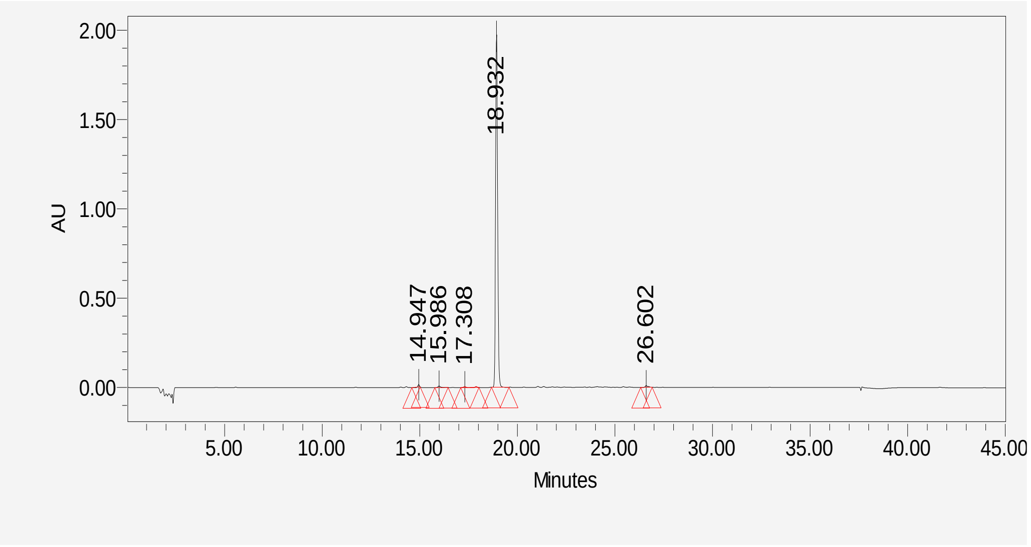

Name	Retention Time	Area	% Area	Height
1	14.947	90044	0.6	13625
2	15.986	83317	0.56	9391
3	17.308	79757	0.54	5558
4	18.932	1.5E+07	97.69	1974991
5	26.602	91579	0.61	9976

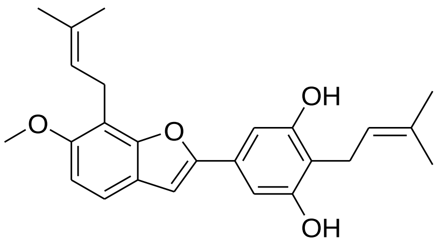

17

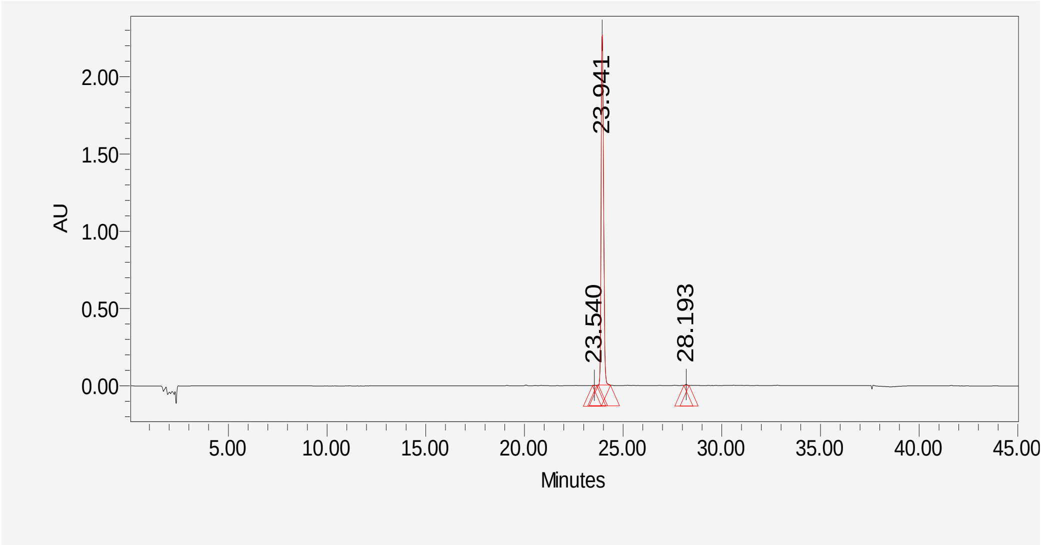

Name	Retention Time	Area	% Area	Height
1	23.54	15490	0.08	2698
2	23.941	2E+07	99.73	2274760
3	28.193	38661	0.2	6100
